# Validity and risk factor analysis for helicopter emergency medical services in Japan: a pilot study

**DOI:** 10.1186/s12873-021-00471-x

**Published:** 2021-07-22

**Authors:** Noriaki Yamada, Yuichiro Kitagawa, Takahiro Yoshida, Sho Nachi, Hideshi Okada, Shinji Ogura

**Affiliations:** 1 Department of Emergency and Disaster Medicine, Gifu University Graduate School of Medicine, 1-1 Yanagido Gifu City, Gifu, 501-1193 Japan; 2 Department of Emergency and Critical Care Medicine, Shimane University Faculty of Medicine, 89-1, Enya-cho, Izumo-shi, Shimane 693-8501 Japan

**Keywords:** HEMS dispatch, Prediction, Validity of dispatch

## Abstract

**Background:**

Some emergency departments use triage scales, such as the Canadian Triage and Acuity Scale and Japan Urgent Stroke Triage Score, to detect life-threatening situations. However, these protocols have not been used for aeromedical services. Therefore, we investigated the factors predicting these life-threatening situations in aeromedical services as a pilot study for establishing the protocol.

**Method:**

We retrospectively evaluated helicopter emergency medical service cases from 1 April 2015 to 31 March 2020 at Gifu University Hospital using the mission records. We only evaluated cases dealing with suggested internal medicine issues. We excluded cases influenced by external factors such as trauma or cases that included hospital-to-hospital transportation, focusing only on prehospital care. We evaluated the validity of the medical emergencies based on the needs for emergency interventions and hospital admission and of the suggested diagnoses and associated risk factors.

**Result:**

A total of 451 cases were suitable for inclusion in the study. In the analysis for all emergency calls, 235 (52.11%) cases needed emergency intervention and 300 (64.4%) required hospital admission. The suggested diagnosis was valid for 261 (57.87%) cases. After the first assessment by emergency medical technicians, 75 cases were removed. Analysis after this first assessment found that 52.31% cases required emergency intervention, 70.26% needed admission, and the suggested diagnosis was valid for 69.41% of cases. In the analysis of emergency calls, the multivariate analysis of some key variables identified age, playing sports, and gasping as risk factors for emergency intervention. Hospital admission risk factors included being age only. The suggested diagnosis was valid only for sports situations. In the analysis after the first assessment by an emergency medical technician, risk factors for emergency intervention included being age being male, playing sports, and gasping, and those for hospital admission was being age, being male, and experiencing stroke symptoms and/or disturbance of consciousness. The suggested diagnosis was valid only for sports situations.

**Conclusion:**

Some ‘second’ keywords/phrases predict medical emergencies. Therefore, the dispatch commander should gather these keyword/phrases to assess.

**Supplementary Information:**

The online version contains supplementary material available at 10.1186/s12873-021-00471-x.

## Background

In medical diagnoses, ‘killer words’ refer to words/phrases that predict life-threatening and serious deceases, such as the sudden onset of chest pain, hemiplegia, unconsciousness, or collapsing. In the emergency department, if the triage nurse/resident meets these words/phrases, these patients’ evaluations and treatments are prioritised compared with other patients in the emergency room. However, we do not decide the triage level depending only on the keyword. Rather, we determine it using patients’ statements and medical history. For example, there are tools for clinical decision making, such as the Canadian Triage and Acuity Scale (CTAS) [1]. This scale is used for the first triage, which is decided based on the patients’ situations and symptoms. For prehospital care, Prehospital CTAS [2] scores are used in North America. As another example, the Japan Urgent Stroke Triage (JUST) score [3] has been used for transport decisions in stroke care. These scoring systems can help paramedics classify patients with suspected stroke.

On the other hand, keyword responses are used for helicopter emergency medical service (HEMS) dispatch in Japan. This means that emergency medical communication centre operators dispatch HEMS according to these killer words. Therefore, we set up key phrases or killer words in the theory book for the operators. This system allows for rapid operator responses. However, the operators’ responses have an increasing probability of failure. The response speed is important, but the dispatch’s validity should be improved because HEMS is a scarce resource.

However, very few studies investigated the accuracy of the HEMS order. We explored some studies regarding trauma dispatch and identified their criteria for selection/triage [4, 5]. In addition, we identified their various dispatch criteria/strategies for specific statuses and diseases [6, 7]. However, no studies systematically investigated the validity of the dispatches in ‘general’ or ‘universal’ orders.

Therefore, we evaluated the validity of keyword dispatches in this study. In addition, by reviewing the records, we examined the trends of these predictive terms under specific situations and evaluated previous records to improve our research quality and prediction of keyword phrases. We believe this study’s results will be helpful to establish protocols and decision strategies for HEMS dispatch.

In the future, we expect to establish a commander-assist scale and systems such as the JUST score. We performed this study as a pilot study to use its results to inform these future studies.

## Materials and methods

This is a single-centre, retrospective observational study. We evaluated the cases of the operated HEMS (Doctor-Heli™) from 1 April 2015 to 31 March 2020 at Gifu University Hospital using mission records from the national database registry project, called J-HEMS. All mission data records are stored as part of the national registry. Using these data records, we focused on keywords related to chest and back pain to predict cardiovascular diseases, sudden onset of hemiplegia predicting stroke, collapsing/unconsciousness predicting cardiopulmonary arrest, and any other internal medicine statuses to order HEMS cases. We excluded cases that suggested trauma or other external causes such as heat stroke. Thus, we focused on the suggested internal medicine emergency cases. In addition, we only focused on prehospital care and excluded hospital-to-hospital transportation cases. We also excluded cases that were not suitable for analysis, for example, patients with congenital diseases.

We evaluated the validity of medical emergencies through the needs for emergency intervention and hospital admission. In addition, we evaluated the validity of the suggested diagnoses. To examine the characteristics of each step, we evaluated the emergency validity in the first dispatch, and the second was examined after being assessed by an emergency medical technician (EMT). This is because if the patient status was not suitable for a HEMS response, the HEMS order would be cancelled.

We evaluated the validity from three viewpoints: need for emergency intervention, need for admission to hospital, and validity of the suggested diagnoses. Then, we evaluated the risk factors for each viewpoint by performing a multivariate logistic regression analysis including predictor variables comprising phrases from the order summary (i.e., age, gender, situation, symptoms, and other characteristics) and dependent variables including needs emergency intervention, hospital admission, and validity of the suggested diagnoses.

### Doctor-Heli™ in Japan

In Japan, the HEMS, called Doctor-Heli™, is organised by the government. Generally, each prefectural government body organises and financially manages the HEMS while being supported by the national government. This means that the public government takes the responsibility for this air ambulance system.

However, the actual operation of the HEMS is assigned to each hospital. At the end of 2018, 43 public bodies organised HEMS, and 53 aircrafts/helicopters were in use in Japan. Gifu University Hospital is one of these assigned hospitals. This hospital’s HEMS covers Gifu prefecture and some parts of neighbouring prefectures. The HEMS operation started in February 2011, and 4252 operations were performed until 31 December 2020. Annually, approximately 500–600 operations are performed at this hospital; approximately 50% of the operations are for prehospital care, approximately 40% are transported to advanced care, and approximately 10% are cancelled. In Japan, the patients and their families cannot directly call Doctor-Heli™.

When emergency medical communication centres receive emergency calls, if the operator deems it necessary to call Doctor-Heli™, the dispatch commander orders a Doctor-Heli™ mission. This operator is a fire department staff member but is not trained systematically.

Each operating hospital has a set call strategy for orders. Each organising body/facility has a keyword list. The keywords have similarities, but there are also differences depending on their situations. The keyword list for Gifu University Hospital and the prefecture is presented in Table 1.

**Table 1:** Keyword lists for helicopter emergency medical service dispatch in Gifu prefecture

Keywords for HEMS orders	
*1st line: If the tele-operator hears the following words, they should order Doctor-Heli immediately*	
1) Keywords that suggest severe trauma	
Motor vehicles accident: locked in	
Motor bicycle accident	
Pedestrian/bicycle traffic accident (hit by a motor vehicle)	
Fall injury (higher than the 3rd floor)	
Traumatic asphyxiation to being buried	
Mass casualty incident	
**2) Keywords that suggest a cardiovascular event or a respiratory disorder**	
**Sudden onset of:**	
**Chest pain**	
**Chest and back pain**	
**Dyspnea**	
**(Patients are normally over 40 years old)**	
**3) Scenarios that commonly suggest a cardiopulmonary arrest**	
**Collapse**	
**Unconsciousness**	
**Respiratory arrest**	
**Pulseless**	
**Convulsions**	
*2nd line: If a dispatcher recognises the following status order they should call Doctor-Heli immediately/if a tele-operator recognises the following emergency words, they should also order Doctor-Heli immediately*	
**1: Consciousness disorder, dyspnea, pallor due to bleeding**	
**2: Shock status**	
**3: Chest pain/chest and back pain**	
**4: Consciousness disorder**	
**5: Status epilepticus hemi-paralysis**	
6: Locked in somewhere for more than 20 min	
7: Fall from the upper level of a building	
8: Hit by a motor vehicle going at a speed of over 30 km/h	
9: Severe burn	
10: Electric injury (including lighting injury)	
11: Mass casualty incident	
**12: Any other stays life threating situations**	

Note: This study focused only on cases with suggested internal medicine situations (bolded part)

### Statistical analysis

Fundamental statistics were obtained from observation data, which were calculated using Microsoft Excel for MAC ver. 16.45. Multivariate analysis was performed using SPSS (IBM).

### Ethical considerations

This study was performed as part of the national database registry project called J-HEMS using the project’s data and Gifu University Hospital’s medical records. This study was approved by the institutional ethical review board of Gifu University/Gifu University Hospital (Medical Review Board of Gifu University Graduate School of Medicine, approval No. 2020–175). Informed consent of the recorded patient was obtained by opt-out on the website and notification in the hospital. Those who rejected this were excluded. In addition, we were given permission to use Gifu University Hospital’s institutional data from the Japan Society for Aeromedical Services.

## Results

**Fig. 1:**
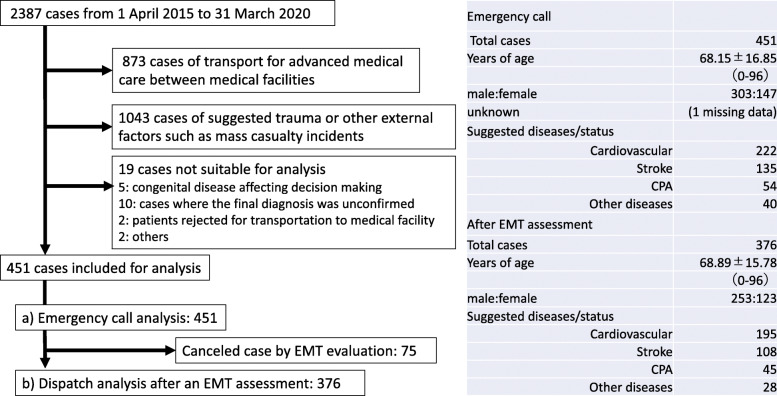
Data collection flow and geographic data

Gifu University Hospital had 2387 recorded cases from 1 April 2015 to 31 March 2020. We excluded 873 cases of transport between hospitals for advanced medical care; 1043 cases of suggested trauma, other external factor diseases, and mass casualty incidents; and 19 cases that were judged unsuitable for analysis, such as cases with congenital diseases affecting decision making and data insufficiency. As a result, 451 cases were included for emergency call analysis, and 376 cases were included for analysis after the first assessment by an EMT. The details and demographic data are shown in Fig. 1.

### Analysis of the validity of HEMS orders

We evaluated the HEMS orders’ validity from three viewpoints: need for emergency intervention, need for admission to hospital, and validity of the suggested diagnoses. Details are shown in Table 2.

**Table 2:** Initial diagnosis validity for each factor

	Ordered		Needs emergency intervention	Needs hospital admission	Validity of the suggested diagnosis
Total cases		451	235	300	261
Only emergency call	(%)		52.10643016	66.51884701	57.8713969
Cardiovascular		222	102	137	124
%			45.94594595	61.71171171	55.85585586
Stroke		135	82	98	74
%			60.74074074	72.59259259	54.81481481
CPA		54	41	44	43
%			75.92592593	81.48148148	79.62962963
Other diseases		40	10	21	20
%			25	52.5	50
Toal Cases		376	235	300	261
After first assesment by an EMT	(%)		62.5	79.78723404	69.41489362
Cardiovasclular		195	102	137	124
%			52.30769231	70.25641026	63.58974359
Stroke		108	83	99	75
%			76.85185185	91.66666667	69.44444444
CPA		45	41	44	43
%			91.11111111	97.77777778	95.55555556
Other diseases		28	10	21	20
%			35.71428571	75	71.42857143

Note: *EMT* emergency medical technician; *CPA* cardiopulmonary arrest

In the analysis of all emergency calls (451 orders), 52.11% needed emergency intervention, 64.4% needed admission, and the suggested diagnosis was valid for 57.87% cases.

When evaluating the suggested disease group, for suggested cardiovascular diseases, 45.95% needed emergency intervention, 61.71% needed admission, and the suggested diagnosis was valid for 55.86% cases. For suggested strokes, 60.74% needed emergency intervention, 72.59% needed hospital admission, and the suggested diagnosis was valid for 54.86% cases. For suggested cardiopulmonary arrest (CPA), 75.93% needed emergency intervention, 81.48% needed hospital admission, and the suggested diagnosis was valid for 79.63% cases.

### Analysis after the first assessment by an EMT

After the first assessment by an EMT, 75 cases were removed from the analysis. Subsequently, we conducted a second analysis. The details are shown in Table 2. The results show that for after the first assessment by an EMT, 52.31% required emergency intervention, 70.26% needed hospital admission, and the suggested diagnosis was valid for 69.41% of cases. When evaluating the suggested disease group, for suggested cardiovascular diseases, 52.31% needed emergency intervention, 70.26% needed hospital admission, and the suggested diagnosis was valid for 63.59% of cases. For suggested strokes, 76.85% needed emergency intervention, 91.67% needed hospital admission, and the suggested diagnosis was valid for 69.44% cases. For suggested CPA, all percentages are quietly high: 91.11% needed emergency intervention, 97.78% needed hospital admission, and the suggested diagnosis was valid for 95.56% cases.

### List of initial diagnoses in the hospital

The list of suggested cardiovascular disease or stroke cases  and the results are presented in Table 3.

**Table 3:** Initial diagnosis of suggested cardiovascular disease and stroke cases that were transported to emergency rooms

Analysis of all emergency calls				Analysis of calls after the first assesment by an EMT			
CARDIOVASCULAR DISEASES	SUSPECTED = 222		%	CARDIOVASCULAR DISEASES	SUSPECTED = 195		%
Cardiovscluar Diseases		124	55.9	Cardiovscluar Diseases		124	63.6
ACS		51	23.0	ACS		51	26.2
	AMI	46	20.7		AMI	46	23.6
	Unstable AP	5	2.3		Unstable AP	5	2.6
AP/non ACS		17	7.7	AP/non ACS		17	8.7
AAD		25	11.3	AAD		25	12.8
Aortic aneurysm rupture		3	1.4	Aortic aneurysm rupture		3	1.5
HF		10	4.5	HF		10	5.1
Arrhythmia		15	6.8	Arrhythmia		15	7.7
Puimonary embolism		3	1.4	Puimonary embolism		3	1.5
Intestinal diseases		6	2.7	Intestinal diseases		6	3.1
Hepatic/bailiarl/pancreatic		4	1.8	Hepatic/bailiarl/pancreatic		4	2.1
Neoplasm		2	0.9	Neoplasm		2	1.0
Phumothrax		1	0.5	Phumothrax		1	0.5
Traumatic		7	3.2	Traumatic		7	3.6
Non specific disorders		24	10.8	Non specific disorders		24	12.3
Others		27	12.2	Others		27	13.8
Canceled caces		27	12.2				
**STROKE**	**SUSPECTED = 135**		**%**	**STROKE**	**SUSPECTED = 108**		**%**
Stroke		75	55.6	Stroke		75	69.4
	ICH	30	22.2		ICH	30	27.8
	CI	27	20.0		CI	27	25.0
	Stroke (not stated)	2	1.5		Stroke (not stated)	2	1.9
	SAH	13	9.6		SAH	13	12.0
	TIA	2	1.5		TIA	2	1.9
Convulsion		7	5.2	Convulsion		7	6.5
Metabolic diseases		4	3.0	Metabolic diseases		4	3.7
Accidental hypothermia		2	1.5	Accidental hypothermia		2	1.9
Heat stroke		2	1.5	Heat stroke		2	1.9
Sepsis		2	1.5	Sepsis		2	1.9
Poisoning		1	0.7	Poisoning		1	0.9
CSDH		1	0.7	CSDH		1	0.9
No disorder		1	0.7	No disorder		1	0.9
Others		14	10.4	Others		14	13.0
Canceled cases		27	20.0				

Note: The diagnoses were collected from medical records. *ACS* acute coronary syndrome; *AP* angina pectoris; *AAD* acute aortic dissection; *HF* heart failure; *ICH* intra cranial haemorrhage; *CI* cerebral infarction; *SAH* subarachnoid haemorrhage; *TIA* transient ischaemic attack; *CSDH* chronic subdural haemorrage

#### Cardiovascular diseases

In the analysis of emergency calls, 55.9% of all cases were cardiovascular diseases. Acute coronary syndrome (ACS) was diagnosed in 23.0% of all suggested cases, and 41% of diagnosed cardiovascular cases. In addition, 12.7% of all suggested and 22.8% of all diagnosed cases were aortic diseases. In the analysis after the first assessment by an EMT, 63.6% were cardiovascular diseases. ACS accounted for 26.2% of all suggested cases, and 13.8% of all suggested cases were aortic diseases.

#### Stroke

In the analysis for emergency calls, 55.6% of all suggested cases were strokes. Intracranial haemorrhage (ICH) occurred in 22.2% of all suggested cases, and 40% were diagnosed stroke cases. Moreover, 21.5% of all suggested, 38.6% of all diagnosed cases were ischaemic stroke, and 9.6% of all suggested, 17.3% of all diagnosed cases were subarachnoid haemorrhages (SAHs).

In the analysis after the first assessment by an EMT, 69.4% were stroke cases. ICH accounted for 27.8% of all suggested cases. In addition, 26.9% of all suggested cases were ischaemic strokes, and 12% of all suggested cases were SAHs.

### Risk analysis

In this study, we analysed factors that affect clinical decisions and outcomes. To reveal which phrases, correspond to which complaints/symptoms, affecting clinical results such as emergency interventions, we analysed various phrases from medical and operation records.

We performed a multivariate logistic regression analysis that included predictor variables comprising some phrases from order summaries (i.e., age, gender, situation, symptoms, and other characteristics) and dependent variables comprising need for emergency intervention, need for hospital admission, and validity of the suggested diagnoses.

In the analysis of the emergency calls, the risk factors for emergency intervention were age the situation of playing sports, and the symptom of gasping. For hospital admission the risk factor was only age. The suggested diagnosis was valid only the situation of playing sports.

In the analysis of the first assessment by an EMT, the risk factors for emergency intervention were being age being male, situation of playing sports, and gasping for air. For hospital admission, the risk factors were being age being male, and experiencing stroke symptoms or disturbance of consciousness. For the validity of the suggested diagnoses, the only risk factor was the situation of playing sports. We also analysed the suggested disease groups for cardiovascular diseases and strokes. The details are shown in Table 4.

**Table 4:** Results of the multivariate analyses for each parameter

Analysis of all emergency calls					Analysis of calls after the first assesment by an EMT				
**Emergency intervention**					**Emergency intervention**				
Variable keyword	OR	95%CI(L)	95%CI(H)	*p*-value	Variable keyword	OR 95%	95%CI(L) 95%	95%CI(H)	p-value
Age	1.105	1.002	1.028	0.024*	Age	1.106	1.001	1.031	0.04*
Male	1.528	0.994	2.351	0.054	Male	1.674	1.029	2.721	0.038*
Situation; under sports	2.32	1.278	4.211	0.006*	situation; under sports	3.387	1.535	7.472	0.003*
Situation; under work	1.356	0.56	3.281	0.5	situation; under work	1.14	0.427	3.309	0.794
Chest pain	0.646	0.377	1.017	0.112	chest pain	0.732	0.406	1.321	0.3
Stroke symptoms	1.131	0.63	2.029	0.68	Stroke symptoms	1.742	0.862	3.521	0.122
Dyspnea	1.071	0.545	2.102	0.843	Dyspnea	1.144	0.533	2.459	0.73
Shock	1.401	0.616	3.186	0.421	Shock	1.149	0.47	2.806	0.761
Gasping for air	3.159	1.083	9.128	0.035*	Gasping for air	8.015	1.009	63.7	0.049*
DOC	1.063	0.62	1.824	0.824	DOC	1.655	0.861	3.183	0.131
Convulsion	0.436	0.103	1.847	0.26	Convulsion	0.634	0.114	3.542	0.604
Emergency call from a healthcare provider	3.099	0.603	15.922	0.176	Emergency call from a healthcare provider	4.188	0.481	36.47	0.195
Emergency call from a family member	0.562	0.128	2.468	0.446	Emergency call from a family member	0.664	0.106	4.154	0.662
**Needs hospital admission**					**Needs hospital admission**				
Variable keyword	OR	95%CI(L)	95%CI(H)	p-value	Variable keyword	OR	95%CI(L)	95%CI(H)	p-value
Age	1.025	1.012	1.039	<0.001*	Age	1.036	1.108	1.055	<0.001*
Male	1.502	0.961	2.347	0.074	Male	1.892	1.056	3.387	0.032*
Situation; under sports	1.642	0.864	3.121	0.13	situation; under sports	0.917	0.917	7.206	0.073
Situation; under work	1.242	0.491	3.139	0.467	situation; under work	0.877	0.279	2.756	0.923
Chest pain	0.661	0.372	1.173	0.157	Chest pain	0.855	0.416	1.754	0.668
Stroke symptoms	1.107	0.582	2.105	0.757	Stroke symptoms	3.209	1.167	8.822	0.024*
Dyspnea	0.923	0.455	1.872	0.823	Dyspnea	0.994	0.405	2.439	0.99
Shock	1.502	0.615	3.67	0.372	Shock	1.276	0.444	3.665	0.651
Gasping for air	2.16	0.675	6.906	0.194	Gasping for air	> 100	< 0.001		
DOC	1.152	0.642	2.067	0.635	DOC	4.19	1.157	11.577	0.006
Convulsion	0.954	0.251	3.626	0.945	Convulsion	6.92	0.538	89.048	0.138
Emergency call from a healthcare provider	3.555	0.417	30.318	0.246	Emergency call from a healthcare provider	> 100	< 0.001		0.999
Emergency call from a family member	0.442	0.097	2.023	0.293	Emergency call from a family member	0.305	0.029	3.194	0.322
**Validity of the suggested diagnoses**					**Validity of the suggested diagnoses**				
Variable keyword	OR	95%CI(L)	95%CI(H)	p-value	Variable keyword	OR	95%CI(L)	95%CI(H)	p-value
Age	1.004	0.992	1.016	506	Age	1	0.985	1.055	0.978
Male	1.134	0.745	1.728	0.558	Male	0.985	0.602	1.16	0.95
Situation; under sports	2.852	1.398	4.468	0.002*	Situation; under sports	4.548	1.842	11.232	0.001*
Situation; under work	1.51	0.623	3.661	0.361	Situation; under work	1.347	0.49	3.7	0.564
Chest pain	0.705	0.411	1.209	0.204	Chest pain	0.752	0.41	0.138	0.358
Stroke symptoms	0.786	0.44	1.406	0.417	Stroke symptoms	0.971	0.485	1.943	0.934
Dyspnea	1.099	0.555	2.178	0.787	Dyspnea	1.135	0.508	2.534	0
Shock	1.331	0.58	3.055	0.5	Shock	1.005	0.406	2.489	0.757
Gasping for air	1.607	0.627	4.119	0.323	Gasping for air	1.603	0.476	5.399	0.991
DOC	0.759	0.444	1.299	0.315	DOC	1.022	5.399	1.692	0.941
Convulsion	0.77	0.215	2.754	0.688	Convulsion	2.615	0.284	24.107	0.366
Emergency call from a healthcare provider	2.74	0.539	13.921	0.224	Emergency call from a healthcare provider	3.308	0.39	28.095	0.273
Emergency call from a family member	0.735	0.173	3.118	0.677	Emergency call from a family member	0.925	0.158	5.433	0.931

Notes: *Signifies statistical significance. *DOC* disturbance of consciousness; *OR* odds ratio; *CI* confidence interval; *EMT* emergency medical technician

#### Analysis for cardiovascular diseases

In the analysis of emergency calls, the risk factors for emergency intervention were being age and situation of playing sports; for hospital admission, they were only being age and male; and for validity of suggested diagnoses, the only risk factor was the situation of playing sports.

In the second analysis, the risk factors for emergency intervention were being age and situation of playing sports; for hospital admission, they were being age and male; and for validity of suggested diagnoses, the only risk factor was the situation of playing sports. Details of the analysis are shown in Supplementary Table 1.

#### Analysis for stroke

In the analysis of emergency calls, the risk factor for emergency intervention was gasping for air, and the downgrade factors were disturbance of consciousness and emergency call from a family member. The only risk for hospital admission was gasping for air. The suggested diagnosis was valid for only gasping for air. The downgrade factor for the validity of the suggested diagnoses was only disturbance of consciousness.

After the first assessment by an EMT, there were no risk factors for emergency intervention and hospital admission. The downgrade factor for validity of the suggested diagnoses was only disturbance of consciousness. Details of the analysis are shown in Supplementary Table 2.

## Discussion

When HEMS are dispatched in Japan, keyword responses are used in many operating hospitals and emergency medical communication centres. This means that emergency medical communication centre operators order HEMS according to the keywords in the list. Each organised body or operating hospital sets up keywords and phrases (killer words) in the theory book for dispatch commanders to ensure rapid responses. However, this system can overestimate and increase the number of unnecessary cases. Therefore, there is a need to develop the theory to guide decisions regarding which cases have high priority. Because HEMS is a scarce resource, we should make the dispatch more effective.

First, we discussed the validity of the HEMS dispatch. It is difficult to define validity. In this study, we set admission as the relation between the suggested diagnosis and initial diagnosis in the hospital, and necessity of admission as ‘correct dispatch’. In addition, as the objectives of the HEMS are providing medical and definitive care as soon as possible, we evaluated possible factors of emergency interventions. No previous studies have evaluated the validity of the dispatch. However, we found symptom-based research on emergency phone protocol. Ellensen et al. [8] investigated emergency medical communication centres’ dispatch resources and transport for stroke patients in Norway. According to their results, the validity of suspected stroke was only 45.6% from the emergency call protocol. Burman et al. [9] investigated data on the epidemiology of acute chest pain outside the hospitals in Norway. They highlighted that the National Advisory Committee for Aeronautics scores indicated that 26% of the patients were in a life-threatening medical situation. Judging from these studies, our analysis of the validity of using keywords theory in our situation when dispatching HEMS is warranted.

However, we believe this validity is insufficient. Because HEMS is a scarce resource, we performed a multivariate analysis with predictor variables being the phrases from order summaries to improve validity. As a result, some keywords were identified as predicting factors. Referring to previous studies, Munro et al. [10] investigated the improvement accuracy of HEMS intervention using an algorithm approach and concluded that when aided by a bespoke algorithm, the accuracy of HEMS dispatch improved. A similar approach is suggested for each symptom and disease group analysis. For example, Pedersen et al. [11] investigated chest pain in acute ambulance transport in the Central Denmark Region and presented its profile and the factors influencing a patient being discharged without a severe cardiac diagnosis and surviving 30 days after a chest pain event. Ellensen et al. [8] investigated emergency medical communication centres’ dispatch resources and transport for stroke patients in Norway and highlighted possible factors associated with stroke prediction. In our study, a similar trend was found. There are possible factors associated with stroke prediction, for example, in an analysis wherein being an elderly male and participating in sports were predictive factors for emergency intervention.

Our results identified the general risk and downgrading factors from the multivariate analysis that were not specific to a patient’s medical history. Therefore, it is not difficult for communication centre operators to gather these medical histories if these risk factors are listed. In fact, Grusd and Kramer-Johansen [12] attempted to analyse whether dispatch triage tools could reliably identify patients who only required transport by analysing electronic and paper records of an ambulance service from four random days in 2012. They concluded that the Norwegian index could predict which patients do not need immediate medical treatment. This study explains the ‘downgrade’ factors, but using predicting systems could also be beneficial. Based on the findings of Grusd and Kramer-Johansen [12], we suggest the following steps.

1. HEMS should be ordered based on the keywords listed in the guidelines.
2. Helicopters take off.
  HEMS personnel stay on the line, while the emergency medical communication centre operators gather a second keyword.
3. HEMS operators receive this information and then score and grade the case to confirm it.

This system could enhance the response speed and decide the priority of each case. Accordingly, it should be investigated in future studies. The final goal is to establish a scoring tool such as the Emergency Department Assessment of Chest Pain Score for ACS [13] to improve the HEMS in Japan.

### Limitations of this study

This single-centre study focused on only one prefecture in Japan. Therefore, the results only reflect the trend of this prefecture and not the Japanese national trend. In addition, we only analysed the order records of one hospital whose information on emergency calls and activities we had access to. Therefore, this information is limited and cannot provide generalisable results. Furthermore, we did not focus on ‘underestimation’ cases. This means that this study did not include cases that were not called in, and hence we could not determine the validity of the cancelled orders.

## Conclusion

As some keyword/phrases can predict medical emergencies, HEMS dispatch commanders should gather these keyword/phrases. Further, we found some trends in HEMS orders. It is thus necessary to perform further analyses using a national database to establish a unified standard protocol for HEMS in Japan.

## Supplementary Information

**Additional file 1.**

**Additional file 2.**

## Data Availability

The datasets generated and/or analysed during the current study are not publicly available. Although this study used a part of the national registry, this registry is not open for public. Further, we were allowed to use Gifu University Hospital’s institutional data by the Japan Society for Aeromedical services, but these data are not open to public access. Nevertheless, the dataset is available from the corresponding author upon reasonable request.

## Abbreviations

AAD: Acute aortic dissection
ACS: Acute coronary syndrome
AP: Angina pectoris
CSDH: Chronic subdural haemorrhage
CI: Cerebral infarction
CPA: Cardiopulmonary arrest
CTAS: Canadian triage and acuity scale
EMS: Emergency medical service
EMT: Emergency medical technician
HEMS: Helicopter emergency medical service
HF: Heart failure
ICH: Intracranial haemorrhage
JUST: Japan urgent stroke triage
SAH: Subarachnoid haemorrhage
TIA: Transient ischaemic attack
