# Supplementary material for: Validity and risk factor analysis for helicopter emergency medical services in Japan: a pilot study
Source: BMC Emerg Med. 2021 Jul 22;21:87. doi: 10.1186/s12873-021-00471-x (PMC8296691; doi:10.1186/s12873-021-00471-x)
Supplement: Supplementary file 1 — Additional file 1. [file 12873_2021_471_MOESM1_ESM.pdf]

Analysis of all emergency calls						Analysis of calls after the first assesment by an EMT					
Emergency intervention						Emergency intervention					
Variable keyword	OR	95%CI(L)	95%CI(H)	p-value		Variable keyword	OR	95%CI(L)	95%CI(H)	p-value	
Age	1.025	1.005	1.046	0.015	*	Age	1.028	1.005	1.051	0.017	*
Male	2.308	1.226	4.344	0.010	*	Male	2.780	1.420	5.442	0.003	*
Situation; under sports	3.345	1.184	9.453	0.023	*	Situation; under sports	5.809	1.512	22.318	0.010	*
Situation; under work	1.604	0.503	5.120	0.425		Situation; under work	1.337	0.407	4.391	0.632	
Stroke symptoms	0.624	0.023	17.235	0.780		Stroke symptoms	0.420	0.013	13.799	0.626	
Dyspnea	1.693	0.710	4.038	0.235		Dyspnea	1.371	0.550	3.418	0.499	
Shock	1.856	0.661	5.212	0.240		Shock	1.556	0.512	4.725	0.436	
Gasping for air	>100	0.000		1.000		Gasping for air	>100	0.000		1.000	
DOC	2.763	0.775	9.847	0.117		DOC	2.010	0.553	7.303	0.289	
Convulsion	>100	0.000		1.000		Convulsion	>100	0.000		1.000	
Emergency call from a healthcare provider	>100	0.000		0.999		Emergency call from a healthcare provider	>100	0.000		0.999	
Emergency call from a family member	N/A	N/A	N/A	N/A		Emergency call from a family member	N/A	N/A	N/A	N/A	
Needs hospital admission						Needs hospital admission					
Variable keyword	OR	95%CI(L)	95%CI(H)	p-value		Variable keyword	OR	95%CI(L)	95%CI(H)	p-value	
Age	1.037	1.016	1.058	0.000	*	Age	1.044	1.019	1.070	0.000	*
Male	1.795	0.962	3.352	0.066		Male	2.456	1.198	5.036	0.014	*
Situation; under sports	2.238	0.736	6.801	0.156		Situation; under sports	4.651	0.919	23.532	0.063	
Situation; under work	1.465	0.432	4.967	0.540		Situation; under work	1.110	0.307	4.014	0.873	
Stroke symptoms	0.278	0.012	6.441	0.425		Stroke symptoms	0.160	0.006	4.268	0.274	
Dyspnea	2.144	0.799	5.754	0.130		Dyspnea	1.653	0.561	4.878	0.362	
Shock	1.609	0.538	4.808	0.395		Shock	1.259	0.371	4.275	0.712	
Gasping for air	>100	0.000		1.000		Gasping for air	>100	0.000		1.000	
DOC	3.972	0.781	20.198	0.097		DOC	2.428	0.470	12.556	0.290	
Convulsion	>100	0.000		1.000		Convulsion	>100	0.000		1.000	
Emergency call from a healthcare provider	>100	0.000		0.999		Emergency call from a healthcare provider	>100	0.000		0.999	
Emergency call from a family member	N/A	N/A	N/A	N/A		Emergency call from a family member	N/A	N/A	N/A	N/A	
Validity of the suggested diagnoses						Validity of the suggested diagnoses					
Variable keyword	OR	95%CI(L)	95%CI(H)	p-value		Variable keyword	OR	95%CI(L)	95%CI(H)	p-value	
Age	1.008	0.990	1.027	0.374		Age	1.005	0.985	1.026	0.607	
Male	0.953	0.528	1.723	0.874		Male	1.024	0.537	1.954	0.942	
Situation; under sports	4.317	1.348	13.828	0.014	*	Situation; under sports	14.435	1.675	124.398	0.015	*
Situation; under work	1.310	0.422	4.065	0.640		Situation; under work	1.132	0.345	3.714	0.838	
Stroke symptoms	0.310	0.011	8.631	0.490		Stroke symptoms	0.147	0.003	7.214	0.334	
Dyspnea	1.567	0.648	3.793	0.319		Dyspnea	1.203	0.472	3.069	0.699	
Shock	1.941	0.682	5.530	0.214		Shock	1.487	0.475	4.654	0.496	
Gasping for air	>100	0.000		1.000		Gasping for air		0.000		1.000	
DOC	2.917	0.737	11.548	0.127		DOC	1.933	0.480	7.789	0.354	
Convulsion	>100	0.000		1.000		Convulsion		0.000		1.000	
Emergency call from a healthcare provider	>100	0.000		0.999		Emergency call from a healthcare provider		0.000		0.999	
Emergency call from a family member	N/A	N/A	N/A	N/A		Emergency call from a family member	N/A	N/A	N/A	N/A	

DOC, disturbance of consciousness
