# Supplementary material for: Validity and risk factor analysis for helicopter emergency medical services in Japan: a pilot study
Source: BMC Emerg Med. 2021 Jul 22;21:87. doi: 10.1186/s12873-021-00471-x (PMC8296691; doi:10.1186/s12873-021-00471-x)
Supplement: Supplementary file 2 — Additional file 2. [file 12873_2021_471_MOESM2_ESM.pdf]

STROKE

Analysis of all emergency calls

Emergency intervention

Variable keyword	OR	95%CI(L)	95%CI(H)	p-value	
Age	0.994	0.967	1.021	0.635	
Male	0.895	0.400	2.001	0.786	
Situation; under sports	0.715	0.246	2.075	0.537	
Situation; under work	1.122	0.143	8.826	0.913	
Chest pain	>100	0.000		1.000	
Dyspnea	3.135	0.289	34.004	0.347	
Shock	0.924	0.047	18.202	0.959	
Gasping for air	21.757	2.383	198.633	0.006	*
DOC	0.325	0.137	0.769	0.010	*
Convulsion	N/A	N/A	N/A	N/A	
Emergency call from a healthcare provider	3.330	0.328	33.790	0.309	
Emergency call from a family member	0.050	0.003	0.919	0.044	*

Needs hospital admission

Variable keyword	OR	95%CI(L)	95%CI(H)	p-value	
Age	1.009	0.981	1.038	0.525	
Male	1.128	0.467	2.721	0.789	
Situation; under sports	0.844	0.270	2.641	0.771	
Situation; under work	0.645	0.086	4.870	0.671	
Chest pain	>100	0.000		1.000	
Dyspnea	1.300	0.124	13.605	0.827	
Shock	>100	0.000		0.999	
Gasping for air	9.539	1.006	90.419	0.049	*
DOC	0.465	0.187	1.154	0.099	
Convulsion	N/A	N/A	N/A	N/A	
Emergency call from a healthcare provider	>100	0.000		0.999	
Emergency call from a family member	0.038	0.002	0.627	0.022	

Validity of the suggested diagnoses

Variable keyword	OR	95%CI(L)	95%CI(H)	p-value	
Age	0.991	0.964	1.018	0.514	
Male	0.744	0.330	1.677	0.476	
Situation; under sports	1.235	0.418	3.650	0.703	
Situation; under work	2.034	0.276	14.972	0.486	
Chest pain	>100	0.000		1.000	
Dyspnea	4.553	0.381	54.403	0.231	
Shock	1.355	0.058	31.801	0.850	
Gasping for air	5.393	1.252	23.226	0.024	*
DOC	0.218	0.088	0.541	0.001	*
Convulsion	N/A	N/A	N/A	N/A	
Emergency call from a healthcare provider	4.752	0.447	50.531	0.196	
Emergency call from a family member	0.000	0.000		0.999	

Analysis of calls after the first assesment by an EMT

Emergency intervention

Variable keyword	OR	95%CI(L)	95%CI(H)	p-value	
Age	0.968	0.927	1.011	0.138	
Male	0.737	0.256	2.128	0.573	
Situation; under sports	0.513	0.134	1.962	0.329	
Situation; under work	>100	0.000		0.999	
Chest pain	0.000	0.000		1.000	
Dyspnea	>100	0.000		0.999	
Shock	0.538	0.027	10.772	0.685	
Gasping for air	>100	0.000		0.998	
DOC	.536	0.180	1.599	0.264	
Convulsion	N/A	N/A	N/A	N/A	
Emergency call from a healthcare provider	1.431	0.139	14.703	0.763	
Emergency call from a family member	0.000	0.000		0.998	

Needs hospital admission

Variable keyword	OR	95%CI(L)	95%CI(H)	p-value	
Age	0.985	0.924	1.049	0.631	
Male	1.096	0.226	5.310	0.910	
Situation; under sports	0.543	0.083	3.538	0.523	
Situation; under work	>100	0.000		0.999	
Chest pain	0.000	0.000		1.000	
Dyspnea	>100	0.000		0.999	
Shock	>100	0.000		0.999	
Gasping for air	>100	0.000		0.998	
DOC	2.126	0.241	18.779	0.497	
Convulsion	N/A	N/A	N/A	N/A	
Emergency call from a healthcare provider	>100	0.000		0.999	
Emergency call from a family member	.000	0.000		0.998	

Validity of the suggested diagnoses

Variable keyword	OR	95%CI(L)	95%CI(H)	p-value	
Age	0.975	0.937	1.013	0.196	
Male	0.670	0.249	1.802	0.428	
Situation; under sports	1.265	0.326	4.907	0.734	
Situation; under work	>100	0.000		0.999	
Chest pain	>100	0.000		1.000	
Dyspnea	>100	0.000		0.999	
Shock	0.847	0.036	19.733	0.918	
Gasping for air	3.010	0.575	15.765	0.192	
DOC	0.308	0.107	0.888	0.029	*
Convulsion	N/A	N/A	N/A	N/A	
Emergency call from a healthcare provider	2.428	0.228	25.845	0.462	
Emergency call from a family member	0.000	0.000		0.999	

DOC, disturbance of consciousness
